# Polycyclic Aromatic Hydrocarbon-degrading Bacteria from Aviation Fuel Spill Site at Ibeno, Nigeria

**DOI:** 10.1007/s00128-012-0598-7

**Published:** 2012-03-29

**Authors:** R. C. John, J. P. Essien, S. B. Akpan, G. C. Okpokwasili

**Affiliations:** 1 Department of Microbiology, University of Port Hacourt, Port Hacourt, Rivers State Nigeria; 2 Department of Microbiology, University of Uyo, Uyo, Akwa Ibom State Nigeria

**Keywords:** Biodegradation, Polyaromatic hydrocarbons, Aviation Fuel-polluted soil

## Abstract

Polycyclic aromatic hydrocarbon (PAH)–degrading bacteria were isolated from aviation fuel contaminated soil at Inua Eyet Ikot in Ibeno, Nigeria. PAH-degrading bacteria in the contaminated soil were isolated by enrichment culture technique. Isolates with high PAH degrading potential characterized by their extensive growth on PAH-supplemented minimal salt medium were screened for their naphthalene, phenanthrene and chrysene degradability. The screening medium which contained selected PAHs as the sole source of carbon and energy showed that *Micrococcus varians* AFS-2, *Pseudomonas putida* AFS-3 and *Alcaligenes faecalis* AFS-5 exhibited a concentration–dependent growth in all the PAH–compounds tested. There were visible changes in the color of growth medium suggesting the production of different metabolites. Their acclimation to different PAH substrates was also evident as *A. faecalis* AFS-5 isolated from chrysene grew well on other less complex aromatic compounds. The isolate exhibited best growth (0.44 OD_600_) when exposed to 10 ppm of chrysene for 5 days and could utilize up to 90 ppm of chrysene. This isolate and others with strong PAH-degrading potentials are recommended for bioremediation of PAHs in aviation fuel-contaminated sites in the tropics.

Polycyclic aromatic hydrocarbons (PAHs) are chemical compounds that consist of fused aromatic rings and do not contain heteroatoms or carry substituents (Fetzer 2000). They are produced during fossil fuel combustion, waste incineration, or as by-products of industrial processes such as coal gasification, production of aluminum/iron/steel and petroleum refining, component of wood preservatives, smoke houses and wood stoves (Shuttleworth and Cerniglia 1995; Gemma et al. 2006). While low-molecular-weight (LMW) PAHs (composed of two or three fused benzene rings) are readily degraded by bacteria, high-molecular-weight (HMW) PAHs consisting of four rings or more are recalcitrant to biodegradation and persist in the environment (Wilson and Jones 1993; Cerniglia 1992).Therefore, the chemical properties and hence the environmental fate of PAH molecules are dependent in part upon molecular size. Generally, an increase in the size and angularity of a PAH molecule results in a concomitant increase in hydrophobicity and electrochemical stability. PAH molecule stability and hydrophobicity are two primary factors which contribute to the persistence of PAHs in the environment. Consequently, they have been detected in numerous aquatic and terrestrial ecosystems at concentrations high enough to warrant concern about bioaccumulation.

Despite the fact that some physical processes such as volatilization, leaching, chemical and photo oxidation which are effective in reducing the environmental level of PAHs (Heitkamp et al. 1988), biodegradation using microorganisms is usually the preferred and major route of PAH removal from contaminated environments because of its cost effectiveness and complete clean-up (Pothuluri and Cerniglia 1994). Besides, the physical processes are often limited to aquatic environments only. Expectedly, PAHs-degrading microorganisms should possess all the necessary enzymes needed to degrade PAHs. However, it is established that biodegradation of PAHs as well as other chemicals occurs as a result of previous exposure to these substances in the environment (Lewis et al. 1984). Adaptation occurs slowly and usually depends on the recalcitrance or biodegradability of the particular substances involved (Spain et al. 1980). This is especially so considering the low aqueous solubility of PAHs which ensures its low bioavailability for microbial utilization (Jonsen et al. 2005). Several species of microorganisms have been successfully utilized in major hazardous waste clean-up processes (Levinson et al. 1994). Abd-El-Haleem et al. (2009) have reported that 2–3 ring PAHs (naphthalene, anthracene and phenanthrene) can be degraded using *Pseudomonas geniculata* and *Achromobacter xylosoxidans*. On the other hand, microbial degradation of high molecular weight (HMW) PAHs including chrysene classified among the priority pollutants by the U.S. Environmental Protection Agency (Smith et al. 1989) is vital to the clean-up of polluted soils and water systems (Alexander 1999; Atlas 1981). Bacterial isolates capable of chrysene metabolism have been described. These include *Rhodococcus* sp. strain UW1 (Walter et al. 1991), *Sphingomonas yanoikuyae* which oxidized chrysene (Boyd et al. 1999) while *Pseudomonas fluorescens* utilised chrysene and benz[a]anthracene as sole carbon sources (Caldini et al. 1995).

There is limited information on microbial degradation of polycyclic aromatic hydrocarbons including chrysene in crude oil-impacted Niger Delta ecosystem. In this work, we report the isolation and characterization of naphthalene, phenanthrene and chrysene-degrading bacteria from aviation fuel contaminated soil in Ibeno, Nigeria.

## Materials and Methods

With the aid of sterile spatula, soil samples were aseptically collected from different location in Aviation fuel contaminated site in Inua Eyet Ikot, Ibeno, Nigeria. All the samples were placed into sterile polythene bags, stored at 4°C in ice-packed coolers and then transported to the laboratory for analysis.

PAH degraders were isolated from soil samples using an enrichment medium containing either naphthalene, phenanthrene or chrysene as sole carbon and energy source (Kastner et al. 1994) The medium contained (per liter): 2.13 g Na_2_HPO_4_, 1.3 g KH_2_PO4, 0.5 g NH_4_Cl, 0.2 g MgSO_4_. 7H_2_O and trace elements solution (1 mL per liter) (Bauchop and Flsiden 1960). This was sterilized by autoclaving at 121°C for 15 min. Thereafter, 0.2 mL acetone solution containing 0.1 % w/v of the selected PAHs (naphthalene, phenanthrene or chrysene) was asceptically pipetted and uniformly spread on the agar surface of a pre-dried plate (West et al. 1984). The acetone was allowed to evaporate under sterile condition before inoculation with 0.1 mL of diluted soil samples. The inoculated plates were covered with foil and black polyethylene bag, and then incubated in the dark at room temperature for 14 days. Control plates free of test PAHs were also prepared and colonies on the control plates were counted and taken as oligotrophs able to grow on the test medium. Colonies that formed crystal clear zones on the PAH-coated plates were replicated onto fresh PAH-coated agar plates and incubated for 14 days. Isolates that grew on these plates were selected as naphthalene, phenanthrene or chrysene degraders.

The isolates were purified repeatedly by sub-culturing and characterized according to procedures described by Cowan (2003) and Holt et al. (1994). The activity of catalase was determined by the appearance of air bubbles after addition of a drop of 30 % hydrogen peroxide solution to an overnight grown single bacterial colony. To determine the ability of isolates to hydrolyze starch, 50 μL of liquid cultures of each isolate were dropped on starch-based solid medium containing per liter, 3 g meat extract, 10 g starch and 15 g agar. The casein hydrolysis was determined by observing zones of clearing after one day of incubation. For this purpose, 50 μL liquid culture of each isolate was dropped on casein-based solid medium containing (per liter) 10 g casein and 15 g agar After one day incubation, the inhibition zones were determined. Carbohydrate fermentation was determined by the production of gas during incubation. The test was performed for fructose, sucrose, glucose, xylose and lactose. Production of tryptophan deaminase was also detected by the color change of the medium after adding 10 % ferric chloride. Glyceride hydrolysis and nitrate reduction tests were also conducted. In addition, the isolates were subjected to Gram’s staining procedure (Harrigan and McCance 1976).

Growth of the different organisms was tested by growing each isolate in a large test tube containing 25 mL of the screening medium supplemented with 15 mg of naphthalene, phenanthrene or chrysene which were dissolved in acetone and added to each tube after autoclaving. Thereafter, the test tubes were incubated at room temperature (28 ± 2°C) for five days. The ability of each isolate to utilize naphthalene, phenanthrene and chrysene was indicated by increase in turbidity of the medium measured at 600 nm using a UV spectrophotometer.

To determine the effect of PAH concentrations on the growth of the isolates, precisely 75 mL of enrichment medium was dispensed into thirty-six 250 mL flasks and sterilized by autoclaving. The flasks were then divided into six sets of six flasks. Thereafter, 50, 100, 150, 200, 250 and 300 ppm levels of naphthalene, phenanthrene and chrysene which were seperately dissolved in acetone (as before) were exposed to each isolate. Inoculated flasks were then incubated as previously described at 28°C for 5 days. Five millilitre sample was aseptically collected from each flask and assayed for the level of microbial growth. Cultures without increase in turbidity over initial optical density (OD) and uninoculated control were scored as no growth (−) while cultures with increased turbidity significantly greater than the control were scored as growth (+), their OD readings were also measured with a UV spectrophotometer.

## Results and Discussion

**Fig. 1:**
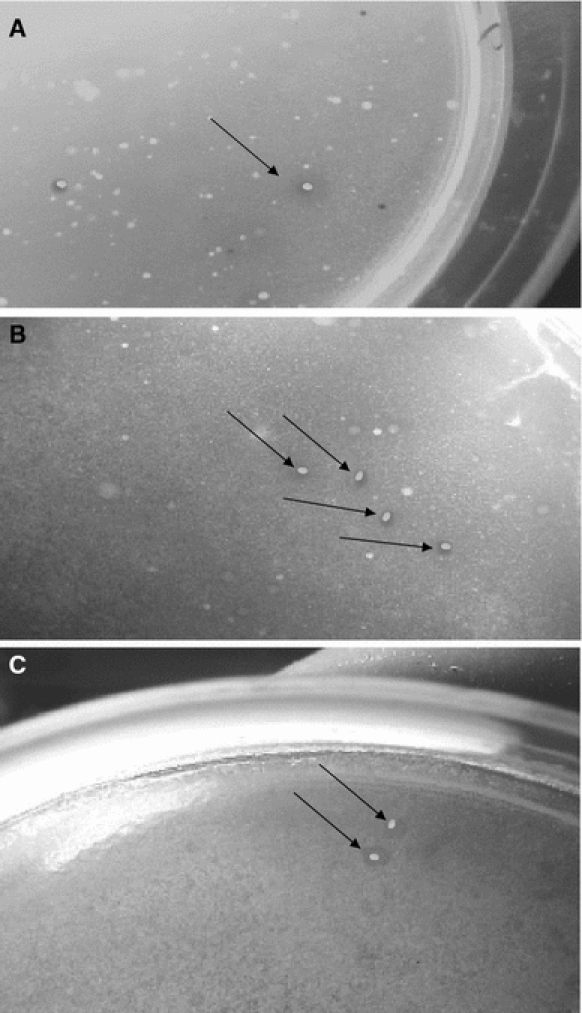
Photograph of the PAH-degrading colonies surrounded by clear zone (*arrow*) of the white coating of PAH **a** naphthalene **b** phenanthrene **c** chrysene

Our findings have shown that aviation fuel contaminated soil is mostly harboured by bacteria with PAH-degrading capabilities (Fig. 1). The PAH-degrading strains isolated from the soil are listed in Table 1. A total of 24 PAH-degrading bacterial isolates were obtained out of which 18 isolates demonstrated the ability to degrade naphthalene. Ten (10) isolates grew on phenanthrene while 14 isolates grew on chrysene (Table 2). Among the naphthalene-degrading isolates, AFS-2 exhibited very high degradability with OD_600_ nm of 0.296. Three isolates, AFS-2, AFS-3 and AFS-5, with high naphthalene-, phenanthrene- and chrysene-degrading capabilities were obtained and characterized. The isolates were respectively identified as *Micrococcus varians* AFS-2, *Pseudomonas putida* AFS-3 and *A. faecalis* AFS-5 (Table 3). Recently, members of the *A. xylosoxidans* capable of degrading PAHs have been isolated from wetland sediment (Wan et al. 2006). *A. xylosoxidans*, first described by Yabuuchi et al*.* (1974), was previously listed under the name *Alcaligenes denitrificans* subspecies *xylosoxidans*, an oligotrophic bacterium. In Nigeria the most common PAH-degrading bacterial isolates are *Pseudomonas*, *Arthrobacter*, *Acinetobacter*, *Flavobacterium*, *Alcaligenes*, *Micrococcus* and *Corynebacterium* (Okerentugba and Ezeronye 2003; Nwachukwu et al. 2000; Ilori and Amund 2000).

**Table 1:** PAH-degradability of bacterial Isolates from aviation fuel contaminated soil

Isolates code	PAH-degradability		
	Naphthalene	Phenanthrene	Chrysene
AFS-1	++	++	++
AFS-2	+++	++	++
AFS-3	++	+++	++
AFS-4	++	++	++
AFS-5	++	++	+++
AFS-6	++	+	++
AFS-7	++	++	++
AFS-8	++	++	++
AFS-9	++	++	++
AFS-10	++	++	++
AFS-11	++	++	
AFS-12	+	++	
AFS-13	++	+	
AFS-14	++	++	
AFS-15	++		
AFS-16	+		
AFS-17	+		
AFS-18	++		

+++, heavy growth; ++, moderate growth; +, weak growth

**Table 2:** Growth performance (optical density) of the PAH- degraders on the different PAHs

Isolate code	Optical density (OD 600 nm)		
	Naphthalene	Phenanthrene	Chrysene
AFS-1	0.133	0.080	0.432
AFS-2	0.296^a^	0.055	0.321
AFS-3	0.114	0.098^a^	0.155
AFS-4	0.068	0.036	0.221
AFS-5	0.082	0.052	0.445^a^
AFS-6	0.134	0.022	0.162
AFS-7	0.084	0.058	0.199
AFS-8	0.068	0.039	0.212
AFS-9	0.042	0.043	0.208
AFS-10	0.089	0.041	0.186
AFS-11	0.098	0.048	
AFS-12	0.02	0.065	
AFS-13	0.026	0.026	
AFS-14	0.110	0.064	
AFS-15	0.042		
AFS-16	0.024		
AFS-17	0.028		
AFS-18	0.033		

^a^Isolates with strong degradability

**Table 3:** Morphological and biochemical properties of the PAH-degrading bacterial isolates

Parameter	*A. faecalis*	*M. varians*	*P. putida*
Gram reaction	–	+	–
Cellular morphology	Rods	Cocci	Short rods
Catalase	+	+	+
Oxidase	+	+	+
Indole	–	–	–
Motility	+	–	+
Methyl red	–	–	–
Voges-Proskauer	–	–	–
Citrate	+	+	–
Urease	–	–	–
Starch hydrolysis	–	–	–
Gelatin hydrolysis	–	+	–
NO_3_ reduction	+	+	+
Coagulase test	–	–	–
Spore test	–	–	–
Mannitol	–	–	–
Glucose	+	+	+
Xylose	–	+	+
Lactose	–	–	–
Sucrose	+	+	–
Arabinose	–	–	–
Maltose	–	+	–
Galactose	+	–	–

**Fig. 2:**
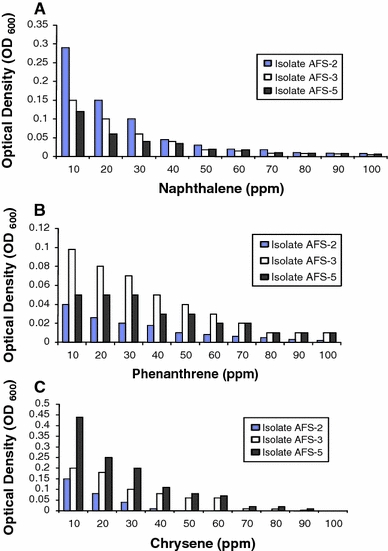
Growth (optical density) of the selected PAH-degrading bacteria on different concentrations of naphthalene, phenanthrene and chrysene. AFS-2 = Naphthalene, AFS-3 = Phenanthrene, AFS-5 = Chrysene

The ability of *M. varians* AFS-2, *P. putida* AFS-3 and *A. feacalis* AFS-5 to degrade naphthalene, phenanthrene and chrysene respectively at different levels of exposure are presented in Fig. 2. For isolate AFS-5, the least growth of 0.01 (OD_600_) was observed at chrysene of 90 ppm while the best growth of 0.44 was recorded when exposed to 10 ppm of chrysene (Fig. 1c) for 5 days. Similarly, the least growth of *P. putida* AFS-3 was 0.015 when treated with 100 ppm of phananthrene, while the highest was 0.098 at 10 ppm of phenanthrene (Fig. 1b). The highest growth of 0.29 (OD _600_) was recorded by *M. varians* AFS-2 treated with 10 ppm of naphthalene (Fig. 1a). However, the growths of all the test isolates were PAH-dependent and provide strong evidence for selective PAH degradation by bacteria. The degradation and utilization of these compounds resulted in increase in optical density (cell mass) of the organisms. However, *M. varians* AFS-2 did not grow well under chrysene concentration range of 40–100 ppm. This inability to utilize the hydrocarbons may be attributed to membrane toxicity and non-possession of the necessary enzymes. Moreso, the lipophilic hydrocarbons accumulated in the membrane lipid bilayer and may affects the structural and functional properties of the membrane. It may also lead to loss of membrane integrity, increase in permeability to protons and consequently, dissipation of the proton motive force, and impairment of intracellular pH homeostasis (Sikkema et al. 1995).

Measuring the success of bioremediation of oil contaminated site is based on several parameters, among them the degradation of polycyclic aromatic hydrocarbons. The three bacterial isolates encountered in the contaminated soil were able to utilize chrysene, phenanthrene and naphthalene. Their ability to utilize both low and high molecular weight PAHs is an indication of the possession of ring fission enzymes (Ilori and Amund 2000). Furthermore, it seems likely that the degradation of individual PAH compounds by the isolates proceeds via independent pathways (Bressler and Fedorak 2000; Van-Hamme et al. 2003). *Alcaligenes faecalis* AFS-5 resisted 90 ppm of chrysene. *M. varians* AFS-2 persisted sparingly in 100 ppm naphthalene respectively. However, the chrysene utilizing bacterium *A. faecalisn* AFS-5 was able to utilize other two tested PAH compounds. It is apparent from the results that strains isolated on chrysene (AFS-5) and phenanthrene (AFS-3) was able to grow better on the three tested PAHs than the strain isolated on naphthalene. These results agree with the observation of Alexander (1999) that the acclimation of microbial community to one substrate, may lead to the simultaneous acclimation to some but not all structurally related molecules. Also, individual microbial species have the ability to act on several structurally similar substrates and therefore more easily act on their analogues after the first addition (Bauer and Capone 1985; Mitchell and Cain 1996).

It is therefore not surprising that *A. faecalis* AFS-5 grew well on these other organic aromatic compounds, considering that they are all commonly composed of benzene rings as phenanthrene (Okpokwasili et al. 1986). Investigation into the regulatory interactions within PAH-degrading consortia and the mechanisms by which HMW PAH biodegradation occur are underway and will prove helpful for predicting the environmental fate of these compounds which is vital to development of practical PAH bioremediation strategies in the future. The degradative capabilities of the evaluated organisms especially *A. faecalis* AFS-5 can be explored in bioremediation strategies for aviation fuel-impacted soil in Nigeria.
